# Beyond dose: an occupational health perspective on architecture as an overlooked component of dental radiology exposure in industrial settings

**DOI:** 10.3389/fpubh.2026.1803266

**Published:** 2026-03-12

**Authors:** Ahmed Adel Mansour Kamar, Ioannis Mavroudis, Alin Ciobica, Diana Gheban, Catalin Morosan, Irina-Luciana Gurzu, Otilia Novac

**Affiliations:** 1 Medical Department, Gulf of Suez Petroleum Company, Gupco, Cairo Main Office, Cairo, Egypt; 2 Department of Orthopedics and Traumatology, Clinical Recovery Hospital (Recuperare), UMF, Iasi, Romania; 3 Department Biology, Faculty of Biology, Doctoral School, Alexandru Ioan Cuza University, Iasi, Romania; 4 Department of Neurosciences, Leeds Teaching Hospitals NHS Trust, Leeds, United Kingdom; 5 Laboratory of Neuropathology and Electron Microscopy, Faculty of Health Sciences, School of Medicine, Aristotle University of Thessaloniki (AUTH), Thessaloniki, Greece; 6 Department of Biology, Faculty of Biology, Alexandru Ioan Cuza University (UAIC), Iasi, Romania; 7 “Ioan Haulica” Institute, Apollonia University, Iasi, Romania; 8 “Olga Necrasov” Center, Biomedical Research Group, Romanian Academy, Iasi, Romania; 9 CENEMED Platform for Interdisciplinary Research, University of Medicine and Pharmacy “Giorge T. Popa”, Iasi, Romania; 10 Faculty of Medicine, Grigore T. Popa University of Medicine and Pharmacy, Iasi, Romania

**Keywords:** cone-beam computed tomography (CBCT), dental radiography, industrial dental clinics, lightweight or artificial wall construction, occupational dental radiation exposure, radiation protection, scatter radiation, thyroid radiosensitivity

## Abstract

Dental X-ray imaging is widely used in daily dental practice and is usually considered safe because radiation doses are low. This belief mainly comes from studies carried out in hospitals and standard dental clinics, where walls and rooms are built with proper radiation shielding. However, dental radiology is not always performed in such environments. In many industrial and corporate workplaces, dental clinics are located inside office buildings that use thin, lightweight, or prefabricated walls. In these settings, wall shielding might be overlooked, absent or not regularly checked. During dental imaging, especially when cone-beam computed tomography (CBCT) is used, scattered radiation may pass outside the dental room, reaching nearby offices or to surrounding working areas. Not only are dental staff exposed, but also other workers who are not considered radiation workers and are not routinely monitored. From this point of view, it is an occupational health perspective based on professional past experiences and published literature. It discusses how building design, wall materials, indoor scatter radiation, and environmental conditions may be overlooked factors in occupational exposure from dental radiology in industrial settings. Special attention is given to the thyroid gland because of its known sensitivity to radiation. No exposure measurements or case investigations are presented, and no causal conclusions are made. The aim of this perspective is to raise awareness and encourage further workplace audits and research on architectural shielding and radiation protection in non-hospital dental clinics.

## 1 Introduction

This article is a hypothesis-generating occupational health perspective informed by professional experience and supported by published literature. It does not present original exposure measurements or epidemiological data.

Dental radiography is an essential part of modern dental practice and is widely considered safe because it uses relatively low radiation doses. This view is mainly based on studies from hospitals and conventional dental clinics, where X-ray rooms are built with proper architectural shielding and controlled radiation areas. Under these conditions, occupational radiation exposure of dental healthcare workers is usually reported to remain well below recommended dose limits (1, 2).

Several studies have shown that occupational radiation doses in dentistry are generally low when standard radiation protection measures are applied. Routine intraoral dental radiography is therefore often seen as low risk, with protection mainly relying on distance, equipment design, and environmental attenuation (2–4). However, occupational exposure can vary depending on imaging technique, workload, operator position, and especially the structural characteristics of the clinical environment in which dental radiology is performed (5).

The safety of dental radiology depends not only on the radiation output of imaging equipment but also on architectural and spatial factors that influence how scattered radiation spreads indoors. In hospital-based clinics, these factors are usually addressed through thick walls, lead-equivalent shielding, and established radiation protection standards. Much less attention has been given to dental clinics that operate outside such conventional healthcare settings.

In many industrial and corporate organizations, permanent medical and dental clinics are located inside non-hospital office buildings. These buildings are often constructed using lightweight, modular, or prefabricated wall materials. In such environments, wall shielding may be absent, insufficient, or not regularly verified. As a result, assumptions derived from hospital-based clinics might not accurately reflect real occupational exposure conditions in these settings.

When dental clinics are built with thin or artificial wall materials, scattered radiation produced during dental imaging may extend beyond the dental operatory and reach nearby offices or workspaces. In these situations, occupational exposure could affect not only dental staff but also administrative employees and other workers who are not classified as radiation workers and are not included in routine radiation monitoring or safety training.

Occupational exposure in dental practice mainly results from scattered radiation rather than direct beam exposure (1, 2, 6). CBCT, which is increasingly used in implantology, orthodontics, and maxillofacial diagnostics, delivers higher radiation doses than conventional intraoral radiography and contributes more to indoor scatter radiation (7–9). Distance from the radiation source and the presence of effective structural shielding remain important factors in reducing occupational exposure (10).

The thyroid gland is one of the most radiosensitive organs in the human body, making long-term low-dose occupational exposure a relevant concern from a public health perspective. Studies of medical radiation workers and exposed populations suggest that repeated or chronic exposure may be associated with thyroid nodules, functional changes, and/or an increased risk of thyroid cancer in genetically predisposed population (11–13). However, most of this evidence comes from settings with well-characterized exposure conditions and standard architectural shielding.

Current radiation protection recommendations in dentistry are possibly based on the principles of justification, optimization, and dose limitation. These guidelines generally assume that dental clinics are designed with adequate wall thickness and appropriate lead or lead-equivalent shielding (1, 14). Such assumptions may not always apply to permanent dental clinics embedded within industrial or corporate buildings constructed with lightweight or modular wall materials.

This perspective is informed by the first author's professional experience as a physician working for several years in a permanent industrial administrative building that housed an on-site dental clinic. During this period, several cases of thyroid disorders were noted among staff working in the same indoor environment. These observations were informal and non-systematic and were not derived from structured epidemiological assessment. No exposure measurements, dose reconstruction, or case investigations were performed. Although these observations raised questions about whether architectural design, indoor scatter radiation, or environmental conditions could represent less frequently discussed contributors to occupational exposure, they served only as hypothesis-generating reflections and should not be interpreted as evidence of causality.

### 1.1 Research question

Do current radiation protection frameworks adequately address the absence of verified wall shielding in long-term dental radiology practice conducted in industrial clinical settings with thin, lightweight, or modular wall construction?

## 2 Methodology

This article is written as an occupational health perspective and not as a systematic or quantitative review. Published scientific literature was used to provide background information and context for the professional observations discussed in this paper.

Relevant publications were identified through general searches of major scientific databases, including PubMed, Scopus, and Web of Science. The literature reviewed focused on occupational radiation exposure in dental practice, scattered radiation from dental imaging, CBCT, radiation protection principles, architectural shielding, and biological effects of ionizing radiation related to thyroid health.

International and national radiation protection guidelines were also consulted to understand current safety assumptions regarding dental radiology, wall shielding, and occupational dose limits (1, 14–19). In addition, reference lists of selected articles were reviewed to identify further relevant publications.

The literature was used to support discussion of architectural and environmental factors that may influence occupational exposure in non-hospital dental clinics. No formal inclusion or exclusion criteria were applied, no systematic appraisal of study quality was performed, and no exposure measurements, case investigations, or statistical analyses were conducted.

The aim of this approach was not to provide an exhaustive review of all available evidence, but to highlight recurring themes and potential gaps relevant to architectural design and occupational radiation exposure in industrial clinical settings.

Key thematic search terms included occupational dental radiation exposure, CBCT scatter radiation, architectural shielding, radiation protection frameworks, and thyroid radiosensitivity.

## 3 Scientific background: dental radiation and occupational exposure

Dental radiography uses ionizing radiation to obtain diagnostic images of teeth and surrounding structures. Common techniques include intraoral radiography, panoramic imaging, and CBCT. Occupational exposure in dental practice occurs mainly through scattered radiation rather than direct beam exposure (1, 2, 6). Although radiation doses in dentistry are generally lower than in other medical imaging fields under standard clinical conditions, occupational exposure may become more relevant in settings where structural shielding is inadequate or radiation safety practices are inconsistently applied (3, 4, 6).

Several studies have shown that occupational doses for dental healthcare workers and other personnel working in proximity to dental imaging areas usually remain below recommended limits when radiation protection measures are properly applied (2, 5). However, exposure levels vary depending on imaging modality, workload, equipment type, and compliance with safety practices.

CBCT, in particular, produces higher radiation doses than conventional dental radiography and contributes more to scatter radiation within the clinic (8, 9). In facilities with lightweight or artificial wall construction, this increased scatter radiation may extend beyond the imaging room, potentially affecting adjacent indoor areas. The main characteristics of common dental imaging modalities and their relevance for occupational radiation exposure are summarized in Table 1.

**Table 1:** Dental imaging modalities and characteristics of occupational radiation exposure.

**Dental imaging modality**	**Common clinical use**	**Relative radiation dose**	**Main source of occupational exposure**	**Key protection measures**
Intraoral radiography	Detection of caries, periapical pathology, routine follow-up	Low	Scatter radiation from patient	Proper operator distance, beam collimation, protective barriers
Panoramic radiography	Evaluation of jaws, impacted teeth, general dental assessment	Low to moderate	Scatter radiation within the imaging room	Structural shielding, operator positioning behind barriers
Cone-beam computed tomography (CBCT)	Implant planning, orthodontics, maxillofacial diagnostics	Higher	Increased scatter radiation during image acquisition	Adequate wall shielding, controlled areas, reduced exposure time, staff training

## 4 Occupational exposure in dental clinics

Occupational exposure in dental clinics is influenced by clinic layout, wall materials, shielding quality, operator position, and the broader architectural context in which dental imaging is performed. Radiation scatter can penetrate surrounding areas if structural shielding is insufficient or improperly designed, as demonstrated even for simple intra-oral dental X-ray units under experimental conditions (6, 12). Standard radiation protection models assume adequate wall thickness and the presence of lead or lead-equivalent barriers, which may not always be the case in non-standard or lightweight clinic constructions (1, 15). In clinics constructed with lightweight or artificial wall materials, such as prefabricated panels or modular structures, scatter radiation may penetrate walls more easily than assumed in standard radiation protection models.

Previous measurement studies have demonstrated that distance from the X-ray source and the use of protective barriers significantly reduce occupational exposure (9). In clinics with limited space or inadequate shielding, dental staff and other personnel working in proximity to dental imaging areas may be exposed to higher cumulative doses, particularly during frequent CBCT use (9).

This risk could be particularly relevant in industrial and corporate healthcare facilities, where dental clinics are embedded within office or field environments and where radiation protection practices might not be routinely audited.

## 5 Mechanistic pathways linking ionizing radiation and thyroid dysfunction

The thyroid gland is discussed here as an illustrative model of a radiosensitive organ to demonstrate biological plausibility and is not intended to represent the sole occupational health outcome of concern.

Ionizing radiation affects biological tissues primarily through the generation of reactive oxygen species (ROS), leading to oxidative stress and potential damage to DNA, proteins, and cellular membranes (10, 11). The thyroid gland is particularly vulnerable to such effects because normal thyroid hormone synthesis physiologically involves oxidative processes, resulting in a higher baseline oxidative burden within thyroid cells (11).

Radiation-induced oxidative stress can contribute to DNA damage, mitochondrial dysfunction, and chronic inflammatory responses in thyroid tissue. Over time, these processes have been associated with thyroid nodules, functional alterations, and an increased risk of malignant transformation in exposed populations with relative variations of genetic predisposition (11, 13). Under conditions of repeated or long-term exposure, even low-dose radiation could therefore have biological relevance, particularly when protective factors such as adequate shielding are possibly insufficient.

### 5.1 Thyroid sensitivity to low-dose ionizing radiation: redox imbalance

The distinctive radiosensitivity of the thyroid gland is closely linked to its intrinsic redox biology. Thyroid hormone biosynthesis depends on hydrogen peroxide generation by dual oxidase enzymes (DUOX1 and DUOX2), which is required for thyroid peroxidase–mediated iodination reactions (20). As a result, thyrocytes function under conditions of sustained ROS production and rely on tightly regulated antioxidant systems to maintain redox balance.

Low-dose ionizing radiation induces additional ROS through radiolysis of intracellular water (21). Although such exposure does not usually cause acute cytotoxicity, repeated or long-term exposure may overwhelm cellular antioxidant reserves and shift the intracellular redox balance toward a pro-oxidative state. This redox imbalance can affect cellular components, disrupt signaling pathways, and impair mitochondrial function, further amplifying oxidative stress within thyroid tissue (22–25).

These mechanisms are discussed to explain biological plausibility only and should not be interpreted as evidence of causation in dental occupational settings, as direct exposure studies in industrial clinical environments are limited.

## 6 Human evidence from occupational and medical radiation studies

Epidemiological studies of medical radiation workers have reported associations between occupational radiation exposure and thyroid abnormalities, including nodules and increased cancer risk (3, 13, 26). An overview of the available human evidence linking radiation exposure to thyroid-related outcomes is summarized in Table 2. Although most evidence derives from settings with better-characterized exposure levels, recent reviews suggest that chronic occupational exposure may affect thyroid structure and function (5, 11).

**Table 2:** Summary of published human studies reporting thyroid-related findings in radiation-exposed populations.

**Study type**	**Population studied**	**Type of radiation exposure**	**Thyroid-related findings**	**Observations reported in the literature**
Narrative and systematic reviews (11–13)	Healthcare and medical radiation workers	Chronic occupational exposure	Thyroid nodules, functional alterations	Long-term radiation exposure discussed in literature as a possible factor that may affect thyroid structure and function
Meta-analyses (3, 26)	Occupationally exposed adults	External ionizing radiation	Thyroid cancer risk	Reported associations between increased thyroid cancer risk with cumulative radiation dose
Cohort and pooled studies (7–9)	Medical radiation workers and exposed populations	Repeated diagnostic or occupational exposure	Structural and malignant thyroid changes	Reported risk estimates vary according to dose, duration, and age at exposure
Dental exposure studies (4, 6)	Dental healthcare workers	Low-dose occupational exposure	Limited direct data	Available evidence remains limited; further focused research is needed

Meta-analyses and pooled studies in the literature have described an increased risk of thyroid cancer following external radiation exposure, with risk magnitude influenced by dose, duration of exposure, age at exposure, and genetic susceptibility (3, 26). Data specific to personnel with occupational exposure to dental imaging remain limited, highlighting the need for further investigation in this occupational group.

## 7 Occupational risk management and regulatory gaps

Radiation protection in dentistry is based on the principles of justification, optimization, and dose limitation (15, 16). Current guidelines emphasize equipment quality assurance, operator training, appropriate positioning, and structural shielding (16). When these measures are applied, occupational doses are generally low.

However, existing regulations largely assume conventional, hospital-grade clinic designs and may not adequately address dental clinics embedded within industrial or corporate facilities constructed with non-standard architectural materials (12, 17, 18). Limited guidance possibly exists on radiation protection requirements for lightweight or modular clinics, where shielding performance might differ from traditional facilities. This represents a potential gap in occupational risk management.

In industrial and corporate settings, dental clinics may operate as permanent healthcare units without being subject to the same level of architectural radiation safety assessment applied in hospital environments. In such facilities, wall construction materials are not always verified for lead-equivalent shielding, and the physical separation between dental imaging areas and surrounding offices may be insufficient. As a result, scatter radiation can extend beyond the dental operatory and affect adjacent indoor workspaces, exposing personnel who are not formally recognized as radiation workers. These structural and organizational shortcomings could represent a potential gap in occupational radiation protection that cannot be mitigated solely through procedural or administrative measures.

Routine radiation safety audits should verify wall materials and shielding performance in non-hospital dental clinics, rather than assuming adequate protection based solely on equipment specifications.

## 8 Discussion and recommendations

From a public health standpoint, even low-probability exposures deserve attention when they involve large numbers of workers over long periods in shared indoor environments. From an occupational health perspective, the main concern raised by this perspective is not radiation dose alone, but the gap between assumed architectural shielding and real-world building conditions in non-hospital dental clinics.

Dental radiographic equipment is generally regarded as safe because manufacturers describe it as emitting relatively low radiation doses in technical specifications and operator manuals. This has contributed to a widespread perception among dental practitioners that dental radiology is safe when used in conventional clinical environments (1, 10). These assumptions are largely based on dental clinics operating within buildings constructed with thick concrete or masonry walls, which provide substantial passive radiation shielding and effective attenuation of scattered radiation (15). Under such conditions, occupational and environmental exposure is typically minimal.

However, this assumption may not apply to dental clinics constructed with lightweight, modular, or artificial wall materials. In these settings, even low-dose scattered radiation can penetrate surrounding structures and extend into adjacent indoor areas. Reliance on nominally low radiation output alone might therefore be insufficient to ensure occupational safety when architectural shielding is inadequate.

Available literature suggests that occupational radiation exposure in dental clinics is influenced by the physical and architectural conditions under which dental imaging is performed (2, 12). While exposure levels are generally well controlled in conventional healthcare facilities with verified structural shielding, this may not be the case in environments with non-standard architectural design. In such settings, limited wall shielding and insufficient physical separation of imaging areas can allow scatter radiation to extend beyond the dental operatory, increasing the potential for unintended occupational exposure (9).

The high radiosensitivity of the thyroid gland, combined with long-term occupational activity in these environments, highlights the importance of prioritizing engineering controls as the foundation of radiation protection (3, 5, 11, 26). Verification of wall construction materials, assessment of lead-equivalent shielding, and appropriate spatial planning of dental imaging areas should be considered essential components of occupational risk management. Procedural measures and radiation safety training, while important, cannot fully compensate for deficiencies in structural radiation protection (15).

Future research should place greater emphasis on evaluating radiation transmission through lightweight or artificial wall materials commonly used in industrial and corporate facilities. Studies assessing indoor scatter distribution and long-term thyroid-related outcomes among personnel working within or adjacent to dental imaging areas are particularly needed to support evidence-based updates to radiation protection guidance (3, 5, 18, 19).

### 8.1 Occupational radiation exposure in permanent industrial healthcare facilities with non-standard architecture

In many industrial sectors, including petroleum and energy industries, permanent medical and dental clinics are integrated within administrative offices and field facilities constructed using lightweight or prefabricated materials. Although these clinics could operate continuously for many years, their architectural design may not incorporate verified lead-equivalent shielding or adequate physical separation between dental imaging areas and surrounding workspaces (12, 17, 18).

As a result, scatter radiation generated during dental imaging procedures can extend into adjacent indoor areas, potentially affecting not only dental healthcare workers but also other personnel who are not formally considered radiation workers (7–9). Existing radiation protection frameworks generally assume hospital-grade construction with inherent structural shielding and may therefore underestimate occupational exposure in these industrial settings (16–18). Addressing this gap requires explicit consideration of architectural design and wall shielding performance in radiation protection guidance for dental clinics operating outside conventional healthcare facilities.

More broadly, the use of portable or mobile X-ray units for occupational health surveillance in industrial settings highlights the need for increased awareness of architectural shielding, spatial planning, and environmental radiation control when imaging is performed outside conventional radiology facilities. These considerations can also be relevant to other forms of occupational imaging performed outside hospital environments, underscoring the importance of preventive design and radiation protection awareness in non-traditional clinical settings.

## 9 Conclusions

Dental radiographic equipment is designed to work at relatively low radiation doses and is generally considered safe when used in properly constructed clinical environments. However, this safety depends strongly on the presence of adequate architectural radiation shielding. When dental radiology is performed in industrial or corporate clinics built with thin, lightweight, or artificial wall materials, these assumptions may not always apply.

This perspective suggests that insufficient wall shielding and limited physical separation of dental imaging areas could allow scattered radiation to extend beyond the dental room. In such situations, exposure may affect not only dental healthcare workers but also other staff working nearby who are not usually included in radiation protection programs.

Relying only on the low nominal radiation output of dental equipment may therefore be insufficient to ensure occupational safety in non-standard building environments.

Overall, this perspective highlights the need to pay greater attention to architectural design and wall construction in radiation protection for dental radiology, especially in long-term industrial clinical settings. Improved verification of shielding, careful spatial planning, and appropriate exposure monitoring could help reduce uncertainty and better align safety assumptions with real working conditions. Further research and possibly development of more advanced workplace safety audits may be needed to better understand occupational exposure in non-hospital environments.

## Data Availability

The original contributions presented in the study are included in the article/supplementary material, further inquiries can be directed to the corresponding authors.
